# Modification of Aerosol Gold Nanoparticles by Nanosecond Pulsed-Periodic Laser Radiation

**DOI:** 10.3390/nano11102701

**Published:** 2021-10-13

**Authors:** Kirill Khabarov, Messan Nouraldeen, Sergei Tikhonov, Anna Lizunova, Alexey Efimov, Victor Ivanov

**Affiliations:** 1 Moscow Institute of Physics and Technology, National Research University, 141701 Dolgoprudny, Russia; messannouraldeen@phystech.edu (M.N.); sergei.s.tikhonov@phystech.edu (S.T.); Lizunova.aa@mipt.ru (A.L.); efimov.aa@mipt.ru (A.E.); 2 Lebedev Physical Institute, Russian Academy of Sciences, 119991 Moscow, Russia

**Keywords:** aerosol nanoparticles, gold, spark discharge, nanosecond pulsed-periodic laser radiation, sintering, shrinkage, densification, plasmon resonance, extinction

## Abstract

This study investigates the processes of interaction of nanosecond pulsed-periodic laser radiation with the flow of aerosol agglomerates of gold nanoparticles synthesized in a spark discharge. Nanoparticles in a gas flow are spatially separated nano-objects whose interaction with each other and with the walls of an experimental cell was insignificant. Therefore, the energy absorbed by nanoparticles was used only for their own heating with further shape and size modification and on heat transfer to the surrounding gas. In the research, we used laser radiation with wavelengths of 527 and 1053 nm at pulse energies up to 900 µJ and pulse repetition rates up to 500 Hz. The dynamics of changes in the nanoparticles size during their sintering process depending on the laser pulses energy is characterized by an S-shaped shrinkage curve. Complete sintering of the initial agglomerates with their transformation into spherical nanoparticles is achieved by a series of impacting laser pulses. The result of nanoparticles’ laser modification is largely determined by the pulse energy and the efficiency of the nanoparticles’ radiation absorption.

## 1. Introduction

The study of optical properties of isolated metal nanoparticles (NPs) of various shapes and sizes is still a challengeable task [1,2]. Until now, the optical properties of completely isolated NPs have been studied by computational methods mainly for simple shapes: for example, for spheres, cylinders, and pyramids [3,4,5], as well as for hybrid core-shell particles [6,7]. The use of such methods to asymmetric particles with a disordered structure is a more complex task, which was solved in a limited number of works, for instance, in [8]. In fact, NPs in liquid dispersions and on substrates inevitably interact with a condensed medium, which can affect their optical properties and thermal regime. These all impose certain restrictions on the experimental research methods. As an example, when studying NPs in colloidal solutions [9,10,11], they are covered with a layer of surfactants or dispersants of the solvent [12,13,14], which contribute to additional energy losses and partial radiation blocking. When studying NPs on substrates surfaces, which is especially interesting for the tasks of laser sintering of NP arrays [15,16,17], the formation of thermal and electrical contacts both between the particles and with the substrate surface [14,16,18] is unavoidable. All of this strongly affects the processes of internal energy distribution in the particles. In such problems, it is also important to take into account possible collective interactions between neighboring NPs that occur at their certain density on a substrate surface [19,20]. All the limitations described above create the need for studying NPs in aerosols free from collective interaction and interaction with a condensed medium.

The optical properties of NPs may be studied in an aerosol stream. The presented method is interesting due to the possibility of on-line measurements of the NPs size and concentration. There have already been attempts to study the properties of aerosol NPs in a gas stream by laser radiation [21]. Until now, the method of irradiation perpendicular to the aerosol flow has remained the main approach for studying NPs characteristics. For example, in [22,23], the authors studied laser-induced incandescence (LII) of soot and iron particles in perpendicular orientation of laser radiation and aerosol flow for investigation of the particles size and their concentration. However, the researchers made no attempts to study NPs by radiation directed along the aerosol flow to obtain a more intense signal with a lower signal-to-noise ratio.

At present, there are several known methods for producing NPs in an aerosol stream with a high purity of the synthesized material. All methods are based on the evaporation of the material and followed vapor condensation and fundamentally differ from each other in the ways of introducing energy for the target material evaporation purposes. These methods include laser ablation [24,25], electrical explosion of wires [26], plasma synthesis [27], and other methods. A spark discharge (SD) method of synthesis is of particular interest [28,29,30,31]. The primary NPs obtained by this method are characterized by a log-normal size distribution with an average size of about 10 nm and a low standard deviation value [32,33], which are comparable to the distributions parameters of NPs obtained by laser ablation [34] and are significantly better than the distribution parameters obtained by other gas-phase synthesis methods.

In this paper, we study the processes of interaction of nanosecond pulsed-periodic laser radiation with wavelengths of 527 and 1053 nm with aerosol gold NPs synthesized by the SD directly in the aerosol stream. For this purpose, we developed a laser modification cell (for simplicity, we will call it just a cell), which combined the studied aerosol flow with the optical axis of impacting laser radiation. In the experiments, the NPs were spatially separated—their interactions with each other and with condensed media (cell walls) were minimized. The study was done at four aerosol flow rates, at which the NPs size distributions differed significantly. In the experiments, we measured the average size and concentration of NPs on-line by their differential electrical mobility depending on the energy and the number of laser radiation pulses. Along with laser exposure, we studied an extinction efficiency of these NPs in a wide spectral range by a spectrophotometer.

## 2. Materials and Methods

**Figure 1:**
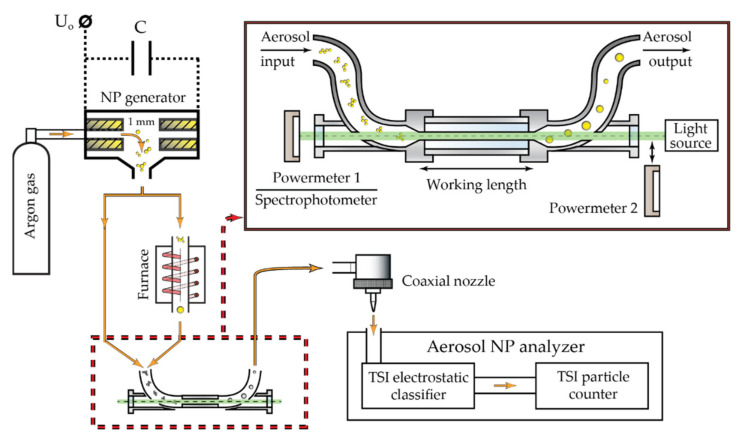
The scheme of the experimental setup: the insert shows a schematic image of the cell combining an aerosol stream and optical radiation, which can optionally be laser radiation or white light.

In this work, the extinction spectra and modification processes of aerosol gold NPs by pulsed nanosecond laser radiation were experimentally studied. Here, it is important to explain that in the following discussion, we will call all the sets of objects formed as a result of the modification of primary NPs agglomerates by laser radiation nanoparticles (also NPs). Aerosol primary NPs were obtained in the SD during the electrical erosion of electrodes in a gas flow. The operation principles of an NPs SD generator with explanations and parameters of the electrical scheme used are described in detail in [28]. Schematically, the entire experimental setup, including the electrical part, thermal modification unit, and optical part for spectral and laser research tasks with the necessary measuring devices, is shown in Figure 1. The electrodes used in the installation were made of gold with a purity of 99.9999% and had a shape of hollow cylinders with an external diameter of 8 mm and a wall thickness of 1 mm. Gold NPs were synthesized in a high-purity argon stream (99.9999%). The high purity of the gold electrodes and the gas medium together with the vacuum tightness of the gas path and synthesis chamber ensured the maximum possible purity of the studied NPs during the experiments.

To study the interaction of optical radiation with aerosol NPs, we used the specially designed and manufactured laser modification cell, which allowed combining an aerosol flow with a laser beam along its length (Figure 1). This device is a vacuum tight U-shaped pipe, which conducts the aerosol flow under an excess pressure of 0.6 bar. The horizontal part of the pipe, combined with the optical axis, is made of a smooth quartz capillary with an internal diameter of 3 mm. Its end faces are equipped with vacuum-tight windows made of Kodial glass, which is transparent in the range of 350–2500 nm. A working length of the cell, in which the aerosol flow is combined with the laser beam, was 145.2 mm. Moreover, the lengths of the curved input and output parts of the aerosol path were much smaller than the working length, and the cross-sections of the gas channel in these parts were significantly widened. This all minimized the contribution of these parts to the studied effects of the radiation interaction with aerosol NPs. Several values of aerosol flow Q = 50, 100, 200, and 400 mL/min were used in the work, which determined the following NPs velocities inside the quartz capillary of the cell: υ = 117.9, 235.8, 471.6, and 943.2 mm/s, respectively.

In some experiments, primary NPs agglomerates were thermally modified in the gas path of a tubular furnace, which maintained a temperature of 750 °C [35] and were located just before the cell. In such processes, agglomerates that passed through the high-temperature zone turned into isolated spherical NPs.

In order to calculate the losses of aerosol NPs due to their deposition on the walls of the aerosol path, we evaluated the efficiency of NPs passing through the cell at all values of the aerosol flow in separate experiments by the laboratory scales SQP-F SECURA 225D-1ORU (Sartorius Lab Instruments, Goettingen, Germany) with a resolution of 10 µg. For this purpose, in a sequential series of two experiments, aerosol HEPA filters were installed in the cell to collect NPs: first before the cell and then after it. In the beginning and at the end of each of the experiments lasting 3 h, filters were weighed to estimate the mass fraction of NPs passed through the cell.

The impact on aerosol gold NPs was studied by nanosecond pulsed laser sources with wavelengths of 527 nm (TECH-527, “Laser-export” Co., Ltd., Moscow, Russia) and 1053 nm (TECH-1053, “Laser-export” Co., Ltd., Moscow, Russia). For these radiation sources, we could control the pulse repetition rate in the range of 10 Hz to 10 kHz with a step of 10 Hz and the pulse energy in the range of up to 180 µJ for the wavelength of 527 nm and up to 900 µJ for the wavelength of 1053 nm. Since the pulse energy adjustment for both lasers could be varied in the full range for pulse repetition frequencies of 10–500 Hz, the experiments were done precisely in these frequencies. At these pulse repetition rates, the pulse duration was about 40 ns for both wavelengths. The lasers were installed alternately at the position of a light source in front of the cell (Figure 1). In all the experiments, radiation was directed opposite to the direction of the aerosol flow. The diameter of the laser beam in the cell was 3 mm for both types of lasers. The studies of the interaction of laser radiation with NPs were done under control of the average radiation power by the thermal power sensor PD300-3W-V1 (Ophir, North Logan, UT, USA) at the input and output of the cell. Using the measured average radiation power $\bar{P}$ and the known pulse repetition rate ν, provided that lasers generate the similar pulses, the energy of one pulse laser radiation was calculated by the formula:

(1) $$E_{pulse}=\frac{\bar{P}}{\nu }.$$

The optical spectra of aerosol NPs in the wavelength range of 350–1000 nm were studied by the spectrophotometer HR4000CG-UV-NIR (Ocean Insight, Orlando, FL, USA) with a white light source (halogen lamp), equipped with an optical fiber, that was installed in front of the cell at the position of the light source. For this purpose, at each flow rate, we did successive measurements of the halogen lamp spectrum through the cell, initially filled only with the gas, and then with the aerosol. The measurements were carried out with the fixed geometry of the experiment and stable position of the optical elements. Final optical spectra of aerosol NPs were obtained by normalizing spectra for the aerosol-filled cell on spectra for the gas-filled cell. This procedure eliminated the influence of possible reflections of laser radiation inside the cell on the results.

Statistical size distributions of aerosol NPs, as well as their concentrations in the gas flow, were controlled after the particles left the cell through a focusing nozzle by the direct measurements of the aerosol NP analyzer based on their differential electrical mobility SMPS 3936 (TSI Inc., Shoreview, MN, USA). This analyzer includes devices: the Electrostatic Classifier 3080—for measuring NPs sizes, and the Ultrafine Condensation Particle Counter 3776—for measuring NPs concentrations. The statistical size distributions of aerosol NPs obtained in such measurements are described by a log-normal distribution. The maxima of these distributions were used to compare the sizes of the initial and modified NPs agglomerates obtained in different regimes as well as to construct the dependences of the NPs modal size on the laser pulses energy and on the pulse repetition period. The focusing nozzle with an outlet diameter of 300 µm simultaneously delivered NPs to the analyzer and stabilized the parameters of pressure and flow of the carrier gas. Additional studies of shapes and sizes of NPs were done based on images obtained by the transmission electron microscope (TEM) JEM-2100 (JEOL, Ltd., Tokyo, Japan). To do this, NPs were collected on TEM grids installed at the nozzle output. According to the obtained TEM images, we collected the statistics of the diameters of modified spherical NPs by processing 600 pieces on average, approximating them with spheres, and plotted their size distribution. As a result of processing, we determined the modal values of distributions, which were used when comparing the sizes of NPs modified in different regimes.

The homogeneity of the NPs concentration along the cross-section of the cell channel is ensured by their effective Brownian motion with velocities in the range of 6–25 mm/s. During the time *t* of the aerosol moving through the cell channel, NPs pass a path $\bar{S_{b}}$ greater than the radius of the channel *R* at all aerosol flow rates:

(2) $$\bar{S_{b}}=\bar{\upsilon_{b}}t=\frac{\bar{\upsilon_{b}}}{\upsilon }L>R,$$

where $\bar{\upsilon_{b}}$ is the average velocity of Brownian motion and *L* is the working length of the cell.

## 3. Results

### 3.1. Characterization of Aerosol NPs

**Figure 2:**
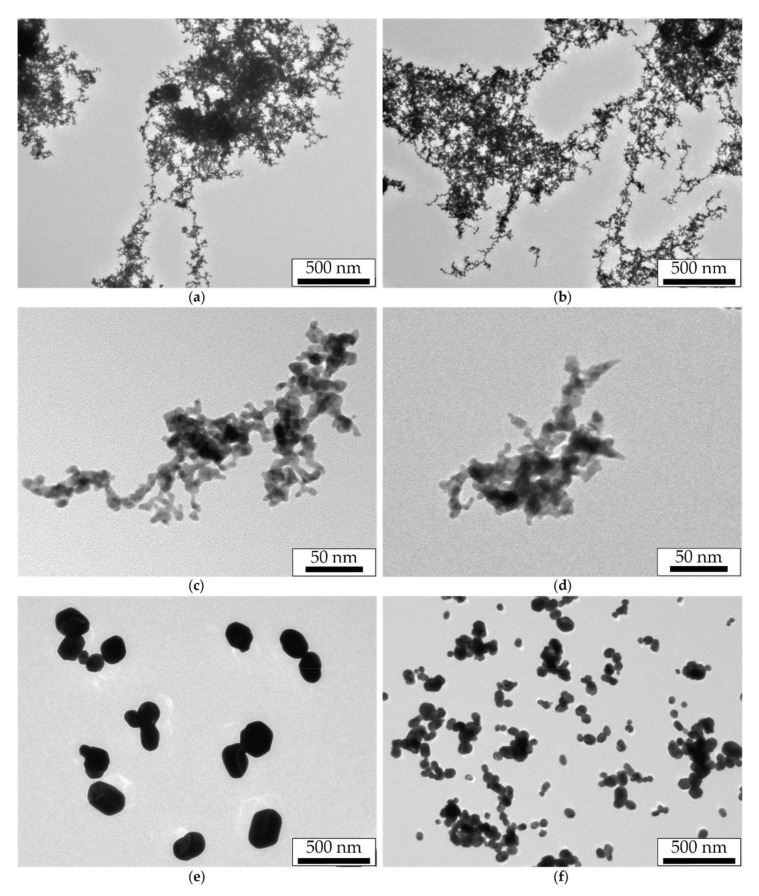
TEM images of (**a**–**d**) gold NPs agglomerates and (**e**,**f**) thermally modified spherical NPs obtained at the flow rates of (**a**,**c**,**e**) 50 mL/min and (**b**,**d**,**f**) 400 mL/min.

The synthesis of primary NPs by the SD is characterized by a high mass production and a narrow size distribution with a maximum of about 10 nm [28]. At the same time, primary NPs during their transportation in the aerosol flow, due to their Brownian motion, experience many collisions and combine into dendrite-like agglomerates of arbitrary shape with an average size of 150–300 nm. Since NPs stay longer in the flow at lower aerosol flow rates, their sizes turn out to be larger in these cases. In our study, the agglomerates sizes measured by the aerosol spectrometer were 280, 240, 233, and 188 nm for aerosol flow rates of 50, 100, 200, and 400 mL/min, respectively. TEM images of agglomerates for the aerosol flow rates of 50 and 400 mL/min are shown in Figure 2a–d.

Another NPs type was obtained by the thermal modification of NPs agglomerates in the tube channel of the furnace. Agglomerates heated to a temperature of 750 °C were modified into spherical droplets (Figure 2e,f) by the mechanism of thermal sintering and then were cooled at the exit of the hot zone. We should note that the transportation of the formed spherical NPs in the furnace channel could lead to their secondary agglomeration due to the increased Brownian motion velocities in the high-temperature zone, leading to an increase in the probability of particle collisions. Obviously, this effect also depends on the gas flow rate, which determines the time spent by NPs in the hot zone, and on their mass and, consequently, size. The brightest result of the secondary agglomeration effect of thermally modified NPs can be seen in Figure 2f.

The sizes of thermally modified NPs were analyzed from a series of TEM images by plotting numerical distributions of the NPs diameter. The average sizes of spherical NPs determined from the obtained data were equal to 183 nm for the gas flow rate of 50 mL/min and 64 nm for the gas flow rate of 400 mL/min. The sizes obtained from microscopy correlate well with the sizes determined by the aerosol spectrometer: for the flow rates of 50, 100, 200, and 400 mL/min, the sizes of spherical particles were 159, 141, 92, and 79 nm, respectively. At the same time, the measured dynamics of the change in the sizes of spherical particles correctly correlates with the change in the agglomerates sizes with an increase in the gas flow rate. Thus, agglomerates with the average size of 280 nm, according to the results of the aerosol spectrometer measurements, at the end of sintering, were transformed into spheres with the average size of 159 nm, and agglomerates with the average size of 188 nm were transformed into spheres with the average size of 64 nm. This corresponded to an increase in the average density of NPs by 5.5 times for the first case and by 25.4 times for the second.

**Figure 3:**
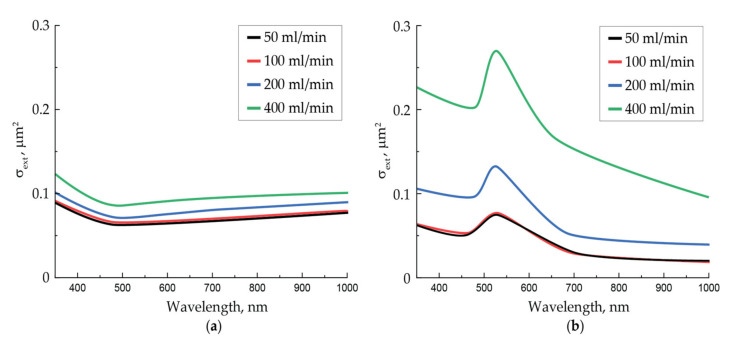
Extinction cross-section spectra of a single NP averaged over the flow of (**a**) initial gold NPs agglomerates and (**b**) thermally modified spherical NPs obtained at different gas flow rates.

For the four NPs synthesis regimes differing in the velocity of the aerosol flow, the aerosol transmission spectra were obtained for both the initial agglomerates and thermally modified NPs. On the basis of these data, we calculated the extinction cross-section spectra of a single NP (Figure 3), averaged over the aerosol flow for particles of each type, according to the Beer-Lambert law [36]:

(3) $$\sigma_{ext}=\frac{1}{nl}\ln\left(\frac{I_{0}}{I_{0}-I}\right),$$

where I and $I_{0}$ are the intensities of the spectrophotometer lamp at a given wavelength at the output of the cell filled with aerosol and with gas only, respectively, *l* is the working length, and n is the concentration of NPs in the gas flow at a given flow rate, as determined by the particle counter.

In general, the measured extinction cross-sections of aerosol NPs are about 10^−13^ m^−2^, which is in good agreement with the results of other authors, presented, for instance, in [37,38]. At the same time, for all gas flow rates, the extinction cross-sections spectra differ slightly for agglomerates and strongly for spherical NPs, except for the spectra taken for the gas flow rates of 50 and 100 mL/min, for which they are practically equal. Probably, this coincidence may be explained by a slight difference in size for large NPs at these flow rates. Since the mass passage efficiency of the cell, determined in experiments with filters, was 97% in the worst case, we maintain that the absorption value is not related to the NPs deposition to the channel walls. This means that the increase in the extinction cross-section for particles of each type is definitely associated with a decrease in the particle size.

The obtained spectra of the extinction cross-sections for thermally modified NPs averaged over the flow predictably demonstrate a plasmon resonance at the wavelength of about 528 nm. It is important to note that with an increase in the gas flow rate corresponding to a decrease in the diameter of thermally modified NPs (compare the images in Figure 2e,f), the Q-factor of the plasmon resonance increases with an almost unchanged spectral position of the resonance peak. A similar rise in the amplitude of the resonant peak while the size of gold NPs falling was previously noted for diameters larger than 70 nm in the works of other authors [39,40]. Another important feature of the spectra is a background level gradually decreasing in wavelength and in the size of NPs formed in an aerosol stream with a higher flow rate. If the peaks on the spectral curves are logically attributed to the plasmon resonance on spherical NPs, then one can associate the spectral background with the interaction of laser radiation with secondary agglomerates formed from thermally modified NPs. It is the sizes of spherical NPs and their agglomerates, differing greatly at the four aerosol flow rates, that are responsible for the significant variation in the corresponding extinction curves.

### 3.2. Aerosol NPs under Laser Radiation with the Wavelength of 1053 nm

**Figure 4:**
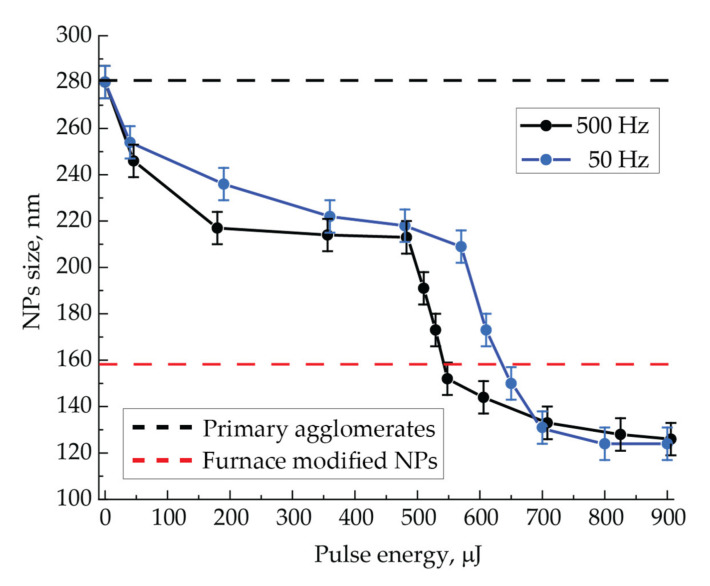
Dependencies of NPs size at the output of the cell on pulse energy of laser radiation with the wavelength of 1053 nm at the pulse repetition frequencies of 50 and 500 Hz and the aerosol flow rate of 50 mL/min.

We studied the effect of nanosecond pulsed-periodic laser radiation impact with the wavelength of 1053 nm on the size of sintered NPs. Their sizes were measured directly in the flow by NPs electrical mobility depending on the pulse energy of incident radiation at the pulse repetition frequencies of 50 and 500 Hz for the aerosol flow rate of 50 mL/min (Figure 4). This figure represents the black and red dashed lines corresponding to the average sizes of initial agglomerates and thermally modified spherical NPs, which are shown here to compare the sizes of NPs modified by laser radiation. Furthermore, we will call the sintering of NPs in the aerosol flow complete if, in the result of their modification, a limit particle size is reached, and it does not change with a further increase in the aerosol temperature in the case of thermal modification, or with the rise of the pulse energy or pulse repetition frequency in the case of laser modification. Otherwise, we consider the sintering incomplete. For example, thermally modified NPs, whose images are shown in Figure 2e,f, should be considered completely sintered, since the energy introduced in this way is sufficient for the formation of spherical NPs [35].

The experimental curves shown in Figure 4 demonstrate a decrease in the size of NPs with an increase in energy of laser pulses. This process should be called an NPs shrinkage in the result of laser sintering in analogy with the shrinkage of macroscopic powder blanks under the energy exposure to them. A typical S-shaped behavior is observed on these shrinkage curves with the increase in input energy. It represents an initially slow decrease in size to a certain value of pulse energy, then a rapid decrease in size in a narrow range of energies and, finally, a slow decrease with an output to a constant value. It is interesting to note that the observed behavior of the shrinkage curves for NPs sintering is also similar to the shrinkage characteristics in the sintering processes of massive powders blanks [41].

The threshold energy value measured at the half-height of the rapid NPs size fall depends on the pulse repetition frequency and amounts to 520 µJ for the frequency of 500 Hz and 640 µJ for 50 Hz. The further asymptotic behavior of the shrinkage curves of about 130 nm corresponds to the complete sintering at the energies transferred to NPs greater than the threshold.

**Figure 5:**
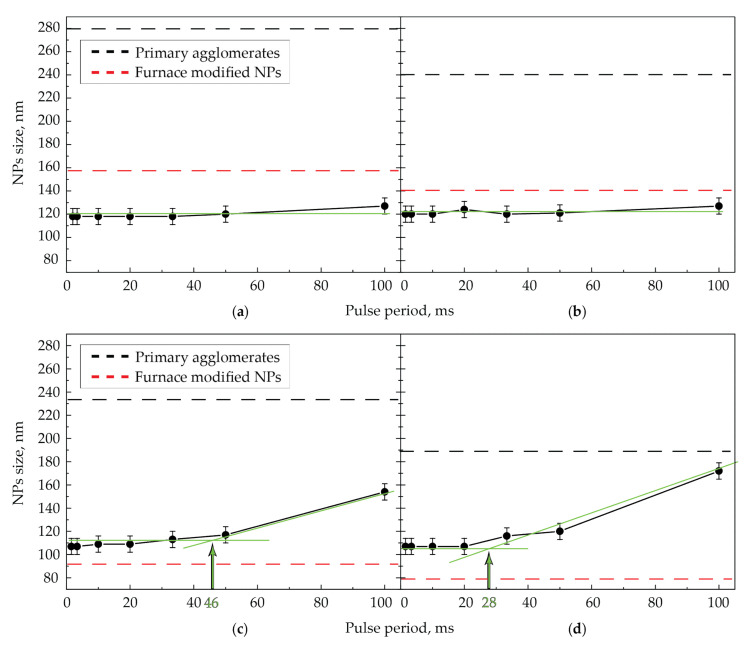
Dependencies of NPs size at the output of the cell on pulse repetition period of laser radiation with the wavelength of 1053 nm at the maximum pulse energy of 900 µJ and the gas flow rates of (**a**) 50, (**b**) 100, (**c**) 200, and (**d**) 400 mL/min.

In another series of experiments, we studied NPs size depending on the pulse repetition period. For that purpose, we used the pulses with a constant maximum energy of 900 µJ and varied the pulse repetition frequency at different aerosol flow rates (Figure 5).

The dependences presented in Figure 5 in most regimes show the complete sintering of NPs in the aerosol flow, as described by the horizontal parts of the experimental curves. At the same time, at low pulse repetition frequencies and high flow rates, the sintering of particles is incomplete, and the curves in these areas have a positive slope (Figure 5c,d). In the process of sintering by infrared (IR) laser at two gas flow rates (50 and 100 mL/min), the sizes of NPs sintered by radiation turned out to be lower than the average size of the NPs modified by the thermal method. This peculiarity might occur due to the absence of the NPs secondary agglomeration effect during their modification by laser radiation, in contrast to the modification of NPs by the thermal method, where the secondary agglomeration of particles was observed (Figure 2e,f).

**Figure 6:**
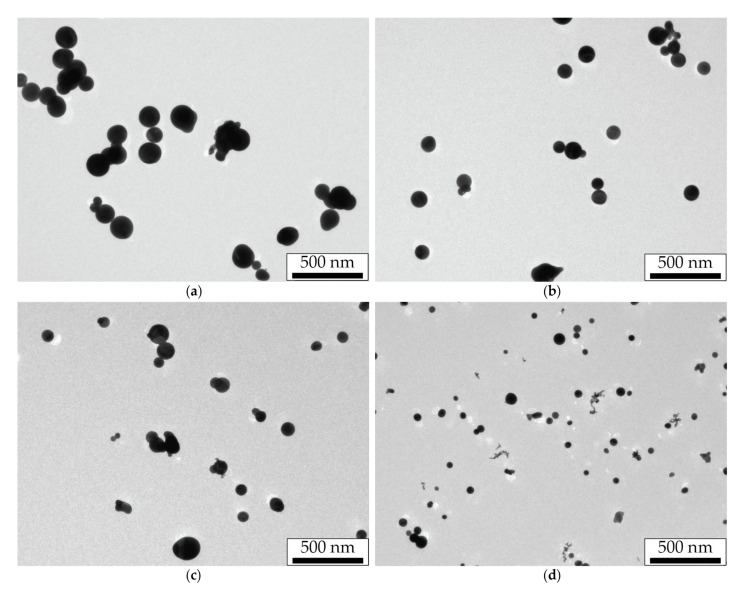
TEM images of gold NPs modified by laser radiation with the wavelength of 1053 nm at the pulse repetition frequency of 500 Hz with the pulse energy of 900 µJ and the aerosol flow rates of (**a**) 50, (**b**) 100, (**c**) 200, and (**d**) 400 mL/min.

The morphology of NPs modified by IR laser radiation at the pulse repetition frequency of 500 Hz with the maximum pulse energy is shown in the TEM images of Figure 6.

These images demonstrate large spherical NPs formed as a result of laser modification of primary NPs agglomerates. The size distributions of sintered NPs were obtained from a series of such images. The corresponding average sizes were 142, 109, 87, and 67 nm for the gas flow rates of 50, 100, 200, and 400 mL/min, respectively. One may notice a strong difference between the sizes obtained from TEM images and from the aerosol NP analyzer for laser-sintered NPs at the gas flow rates of 200 and 400 mL/min. This feature hints at the presence of a significant part of untreated NPs in the composition of these aerosol streams, which is especially clearly confirmed by Figure 6d.

Extinction cross-sections of laser-modified NPs were determined from the measured values of the average power at the input and output of the cell. At the wavelength of 1053 nm at the maximum laser pulse energy and pulse repetition rate of 500 Hz at the gas flow rates of 50, 100, 200, and 400 mL/min, extinction cross-sections of completely sintered NPs by laser radiation $\sigma_{LM}$ are presented in Table 1 in comparison with extinction cross-sections of the initial agglomerates $\sigma_{A}$ and thermally modified NPs $\sigma_{S}$ based on the data from Figure 3.

### 3.3. Aerosol NPs under Laser Radiation with the Wavelength of 527 nm

**Figure 7:**
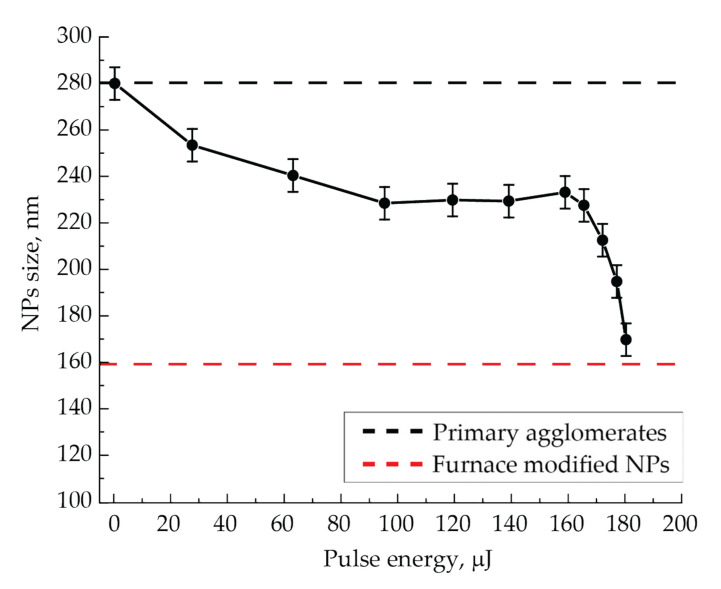
Dependence of NPs size at the output of the cell on pulse energy of laser radiation with the wavelength of 527 nm at the pulse repetition frequency of 500 Hz and the aerosol flow rate of 50 mL/min.

We also investigated the sintering processes of aerosol NPs by pulsed-periodic laser radiation with the wavelength of 527 nm. In the first series of experiments, in analogy with IR radiation, sizes of sintered NPs were measured in the flow by their electrical mobility on the pulse energy of incident radiation for an aerosol flow rate of 50 mL/min at a pulse repetition frequency of 500 Hz (Figure 7). In this figure, the black and red dashed lines, as in Figure 5, correspond to the average sizes of initial agglomerates and thermally modified spherical NPs and are shown to compare sizes of NPs modified by laser radiation.

The NPs sintering curve for laser modification by green laser radiation (Figure 7) clearly demonstrates a typical shrinkage initially with a slow decrease in size and then with a rapid one as the input energy increases. It is quite expected that with a further increase in laser pulse energy, the size of the sintered NPs after reaching the red dashed line will come to saturation, demonstrating the S-shaped behavior of shrinkage curves; i.e., full sintering will be realized. In this case, the sintering curve was obtained by varying the pulse energy up to the maximum value of 180 µJ, so the asymptotic behavior of the curve was not achieved. Such sintering should be considered as incomplete. Nevertheless, the sizes of NPs modified by green laser pulses with the maximum energy were found to be close to the size of the completely sintered NPs by the thermal method.

**Figure 8:**
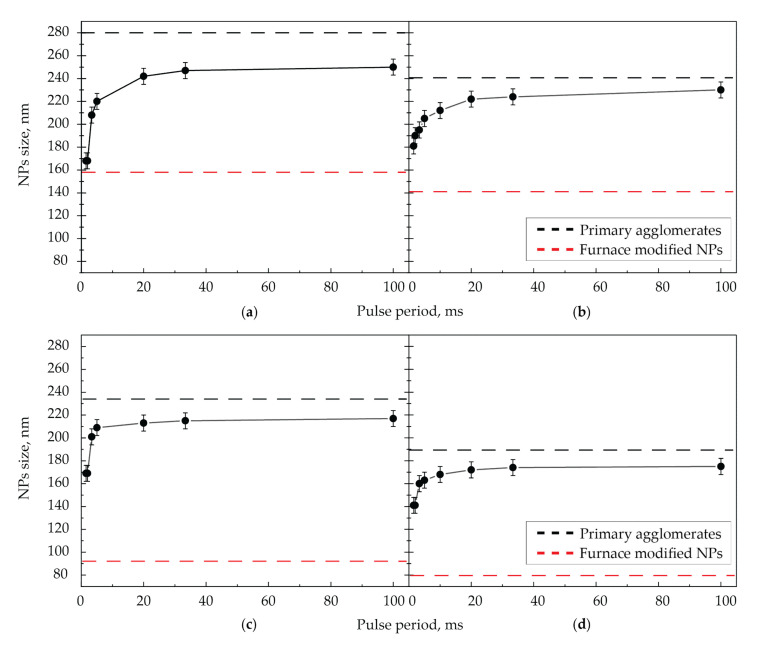
Dependencies of NPs size at the output of the cell on the pulse repetition period of laser radiation with the wavelength of 527 nm at the maximum pulse energy of 180 µJ and the gas flow rates of (**a**) 50, (**b**) 100, (**c**) 200, and (**d**) 400 mL/min.

In the second series of experiments, the size of NPs sintered at the maximum pulse energy of 180 µJ was measured depending on the pulse repetition period for the four aerosol flows (Figure 8). Since the amount of energy absorbed by NPs in these experiments was not higher than in experiments with varying pulse energy, complete sintering of NPs was not expected. However, it was interesting for us to see the dynamics of NPs sintering at the current wavelength depending on the number of pulses impacting on a single NP moving in the cell in analogy with the experiments with the IR laser.

The solid black curves position (the size of laser-modified NPs) under the dashed black lines (the size of initial agglomerates) indicates that the sintering of NPs by laser radiation occurred in all cases. For all values of the aerosol flow in the graphs, as the pulse repetition period increases, a sharp increase in size is initially observed, and then, starting from the pulse repetition period of 10 ms, the NPs size increases slightly. In addition, with an increase in the aerosol flow at the same pulse repetition rates, the modification efficiency decreases.

**Figure 9:**
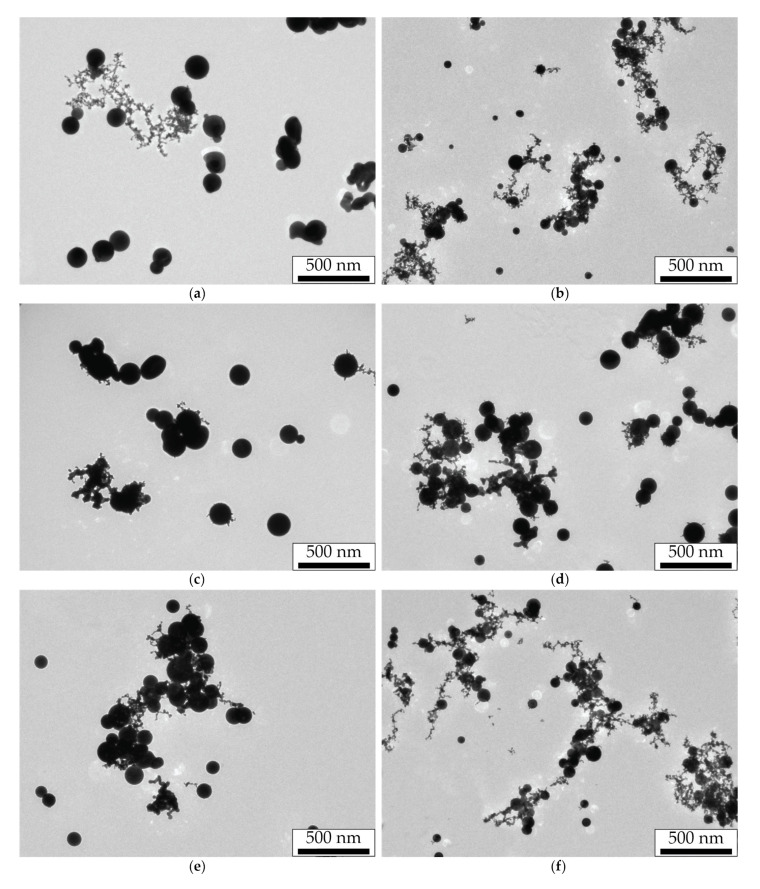
TEM images of gold NPs modified by laser radiation with the wavelength of 527 nm and the pulse repetition frequency of (**a**,**b**) 10 Hz and (**c**–**f**) 500 Hz at the pulse energy of 180 µJ and the aerosol flow rates of (**a**,**c**) 50, (**d**) 100, (**e**) 200, and (**b**,**f**) 400 mL/min.

NPs modified by green laser radiation with the maximum pulse energy at low (10 Hz) and high (500 Hz) frequencies were analyzed in a series of TEM images (Figure 9).

From the analysis of the series of TEM images, the average size of the modified NPs was 160, 100, 92, and 70 nm for the frequency of 500 Hz and the gas flow rates of 50, 100, 200, and 400 mL/min, respectively, and also 142 and 55 nm for the frequency of 10 Hz and the gas flow rates of 50 and 400 mL/min, respectively. The presented sizes of spherical NPs modified by green laser radiation turn out to be close to the sizes of NPs sintered by IR laser radiation. However, smaller particles adjacent to these spheres clearly indicate the incompleteness of the sintering processes in these regimes, which is the main difference. With an increase in the gas flow rate, the incompleteness of sintering becomes more obvious—longer and branched chains of smaller particles are observed around sintered spherical particles, and their appearance becomes more similar to the original dendrite-like agglomerates (Figure 2a,b). Moreover, images of NPs obtained by sintering at different pulse repetition frequencies for the gas flow rate of 50 mL/min differ more strongly than for the flow rate of 400 mL/min.

Measured values of the average power at the input and output of the cell for laser-modified NPs were used to determine their extinction cross-sections $\sigma_{LM}$ at the wavelength of 527 nm at the maximum pulse energy and pulse repetition rate of 500 Hz. For gas flow rates of 50, 100, 200, and 400 mL/min, these values are given in Table 2 in comparison with the extinction cross-sections for the initial agglomerates $\sigma_{A}$ and thermally modified NPs $\sigma_{S}$ based on the data from Figure 3.

## 4. Discussion

Significant differences in spectral characteristics of extinction for initial agglomerates and thermally modified NPs (Figure 2), depending on the aerosol flow rate, can be explained by the difference in the average size of the studied NPs, which increases simultaneously with a decrease in the gas flow rate. Dendrite-like agglomerates interact with light as a whole due to its ramified structure, acting as spatial nanoantennas [42,43]. Thus, they absorb and dissipate light with similar efficiency in the range of 350–1000 nm. Spherical NPs produced by the thermal modification of agglomerates also became bigger with a decrease in aerosol flow rate because their size clearly depends on the size of initial agglomerates. It is the differences in sizes of initial agglomerates and thermal-modified NPs that are the main reason for an increase in the extinction cross-section with an increase in the gas flow rate. Such a regularity was previously observed in [44], where with the enlargement of gold NPs agglomerates, a decrease in their absorption ability in the visible wavelength range and an increase in the far IR range were found. Researchers also previously reported on the similar pattern of a decrease in the absorption efficiency of spherical gold NPs with an increase in their size above the critical value of about 70 nm, which was mainly associated with the conditions deterioration for the excitation of plasmon oscillations [39,40].

Different spectral characteristics of the extinction cross-section of NPs, depending on their size and shape, determine the efficiency of laser radiation energy absorption during the NPs sintering. Since agglomerates absorb green radiation slightly worse than IR, the initial stage of sintering with IR radiation will occur more actively. For example, this is especially clear at low laser pulse energies (the initial course of the curves in Figure 4 and Figure 7), when NPs have begun to decrease in size but are still far from being spherical. During the sintering process, NPs obtain a spherical shape during modification, and as follows from the spectral characteristics, the absorption efficiency of green radiation should increase due to the improved conditions for the excitation of plasmon resonance, and in the IR range, on the contrary, it should fall. However, with the aerosol flow rates in the range of 100–200 mL/min and, consequently, at a certain size of initial agglomerates, one can conclude from comparing the spectra for two types of particles that the initial and final radiation absorption efficiency will be the same, since in these regimes, the extinction cross-sections of the two types of particles are close.

The data on the extinction cross-sections of laser-modified NPs presented in Table 1 and Table 2 characterize their optical properties and level of modification by radiation with wavelengths of 527 and 1053 nm. A much lower extinction cross-section of NPs completely modified by IR radiation, compared with the extinction cross-sections of the initial agglomerates and thermally modified NPs, allows us to exclude the effect of secondary agglomeration of laser-modified NPs, which is characterized by an increased extinction in this wavelength range. This is also evidenced by the NPs TEM images (Figure 2f and Figure 6a–d) obtained in the same regimes. On the other hand, differences in the extinction cross-sections of NPs modified by green radiation confirm the incompleteness of the sintering process observed in TEM images (Figure 9c–f).

The process of sintering NPs by laser radiation in the cell also significantly depends on the time NPs are in the working channel, which is determined by the aerosol flow rate and channel length. The estimated time of NPs flight through the channel with a length of 145.2 mm was 1232, 616, 308, and 154 ms for the aerosol flow rates of 50, 100, 200, and 400 mL/min, respectively. Thus, at the considered aerosol flow rates at a fixed pulse repetition frequency, NPs were modified by a different number of pulses. However, the complete sintering was primarily determined by the amount of energy absorbed by NPs. Therefore, due to the larger regulating range of the laser pulse energy at the wavelength of 1053 nm, the shrinkage during NPs sintering was studied at several pulse repetition frequencies. In this case, a more complete sintering was reached than at the wavelength of 527 nm at the maximum values of the pulse energy of 180 µJ and the pulse repetition frequency of 500 Hz.

An important feature when nanosecond pulsed-periodic laser radiation was applied to NPs in the aerosol stream is that a certain portion of pulse energy was transferred to the nano-object during each pulse. This energy was used for sintering and heat transfer to the surrounding gas long before the next pulse arrived. Indeed, the theoretically estimated cooling time of a spherical gold NP with the diameter of 100 nm in an argon atmosphere is about 50 ns on average [45,46], and the minimum repetition period of laser pulses, equal to 2 ms at the maximum used frequency of 500 Hz, turns out to be significantly longer. Thus, while using nanosecond pulsed-periodic laser radiation, there was no energy accumulation in aerosol NPs, and each subsequent pulse heated the particle anew. This means that when a set of successive radiation pulses is exposed to a sintering NP during its flight in the cell, its shape and size change discretely-stepwise. This happens for short periods of time while the nano-object is heated by pulse impact. Therefore, the conditions for the energy absorption of each subsequent pulse by the same particle differ due to the change in its shape during sintering.

Another fundamental aspect of the NPs agglomerates modification processes by nanosecond pulsed laser radiation is that most of the time, the particles are at the temperature of the surrounding gas, as, for example, at the room temperature in our experiments. Then, the behavior of these particles should correspond to the ambient temperature, as the Brownian motion velocities of the particles modified by laser radiation will be lower than in the case of thermal modification in a hot gas stream. In turn, this means that the effect of secondary agglomeration of NPs modified by laser radiation will be much weaker in comparison with thermal modification.

**Figure 10:**
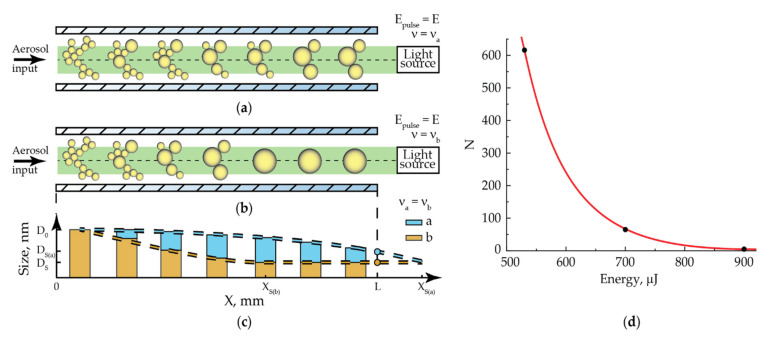
Illustrations of the NPs modification processes in the cell by laser radiation with different repetition rates (**a**) ν_a_ and (**b**) ν_b_ and the equal pulse energies E and (**c**) the corresponding qualitative graph, representing the NPs size along the cell; (**d**) an estimated graph of required number of pulses for complete sintering of NPs for the working length of 145.2 mm on energy of laser pulses for radiation with the wavelength of 1053 nm.

When a sufficiently large laser pulse energy interacts with NPs, their complete sintering can occur. This will require a certain number of pulses due to a discrete-stepwise mechanism for shape and size changing. Such sintering was realized by IR radiation with the pulse energy of more than 700 µJ (Figure 4). Obviously, we should expect a decrease in the number of pulses with an increase in their energy up to one pulse. The number of radiation pulses required for NPs complete sintering at a certain pulse energy can be determined from the experimental data on the NPs shrinkage, in particular, from the data in Figure 5. For its interpretation, let us consider the process of modifying NPs moving through the quartz capillary of the cell, as shown in Figure 10a,b. Here, initial NPs enter the cell on the left. As they move, they are exposed to a certain number of laser pulses, which provides a discrete-stepwise sintering process. At the exit from the cell on the right, we measure the size of partially or completely sintered particles. Thus, particles of various shapes and sizes move in the flow along the working length of the capillary. The illustrations presented in Figure 10a,b clearly characterize this process for two different repetition rates of laser pulses ν_a_ and ν_b_ at their same energy E. The graph shown in Figure 10c qualitatively reflects the sintering process of NPs with the initial size D_0_ by laser radiation pulses. The case with the pulse repetition frequency ν_a_ demonstrates an incomplete sintering process with the final particle size D_S(a)_ at the exit from the cell. In this case, complete sintering could be achieved either at the longer working length X_S(a)_, or at the current length L with a larger number of radiation pulses, for example, by slowing down the aerosol flow or increasing the pulse repetition frequency. The last variant with an increased pulse repetition rate ν_b_ is just shown in Figure 10b,c, which demonstrates the complete sintering of particles up to the size D_S(a)_ = D_S_ at the working length X_S(b)_ < L.

Based on the presented qualitative model, we approximately estimated the minimum number of pulses required for complete NPs sintering by laser radiation with the wavelength of 1053 nm at a fixed laser pulse energy. For that purpose, we used the experimental dependences of particle size on the laser pulses’ repetition period (Figure 5). The complete sintering of NPs was already achieved at such a value of the pulse repetition period, if the particle size at the exit of the cell did not change with the period decrease. Therefore, the number of laser pulses that ensures the complete sintering of NPs was determined by the ratio of the time of aerosol movement along the cell to the set value of the pulse repetition period. Graphically, the value of the pulse repetition period is located at the intersection of two approximations of the dependence of NPs size on pulse repetition period. Namely, these are a horizontal part of the curve corresponding to the size of fully sintered particles and a part of the curve under the slope corresponding to the size of particles with different sintering incompleteness (an example of the intersection is represented by green lines in Figure 5c,d). The approximations were done by straight lines by the least squares method for the ease of interpretation; however, in reality, the inclined section may be nonlinear. In the case of radiation with the wavelength of 1053 nm and the pulse energy of 900 µJ at the aerosol flow rate of 400 mL/min, complete sintering occurred with the pulse repetition period of about 28 ms. With the known time of aerosol movement through the cell of 154 ms at the given flow rate and the graphically defined pulse repetition period, the number of pulses for complete sintering was found equal to 6. Approximately the same number of pulses was calculated from the graphs for the other gas flow rates: at 200 mL/min, it turned out to be 7, and at 100 and 50 mL/min, the upper estimate was 6 and 12 pulses, respectively, since already at the pulse repetition period of 100 ms, the size dependence on the period is horizontal. It is also correct to claim that the gas flow rate affects the number of pulses required for sintering only in the context of different sizes of initial agglomerates but not in the sense of speed parameters. That is, large agglomerates at lower aerosol flow rates may require a slightly larger number of radiation pulses for sintering.

Additionally, the minimum number of pulses required for NPs sintering by IR radiation can be estimated from the dependences of particle size on the pulse energy shown in Figure 4. The pulse energy corresponding to the end of the sharp decline and the beginning of the horizontal part in the dependence of particle size on pulse energy (Figure 4) at the two studied frequencies of 50 and 500 Hz is just enough to conduct a complete sintering at the cell working length L at the aerosol flow rate of 50 mL/min. From these data, the number of pulses required for NPs complete sintering at the defined pulse energies of 540 and 700 µJ turns out to be equal to 616 and 62, respectively.

Based on the data presented above, we can propose an estimated dependence of number of radiation pulses required for complete sintering of gold NPs in the cell with the working length of 145.2 mm on pulse energy. This dependence, as shown in Figure 10d, has the form of a decreasing exponent. From this dependence, it was estimated that for the NPs’ complete sintering by a single radiation pulse with the wavelength of 1053 nm, its energy must be at least 970 µJ.

Similarly, it is not possible to determine the number of laser radiation pulses with the wavelength of 527 nm and the maximum energy of 180 µJ for complete NPs sintering based on our data, since the level of complete sintering was not achieved at the used pulse repetition rates. However, the lower estimate of the required number of pulses for sintering by that radiation is equal to 616 and is calculated as the product of the time of aerosol movement through the cell at the flow rate of 50 mL/min and the maximum pulse repetition rate of 500 Hz.

The conducted experiments allow comparing the method of NPs sintering in an aerosol stream by nanosecond laser radiation pulses with the thermal modification method. As it can be seen from the NPs TEM images (Figure 2e,f and Figure 6), the efficiency of sintering by pulsed laser radiation with the wavelength of 1053 nm at the pulse repetition frequency of 500 Hz and the pulse energy of 900 µJ was not worse than by temperature exposure. At the same time, based on the results shown in Figure 4 and Figure 5, the same effect can be achieved either with the same number of interacting pulses and their lower energy of about 700 µJ or with the much smaller number of pulses of up to 10 with higher energy. Comparing TEM images of particles sintered by the thermal method and by laser radiation with the wavelength of 527 nm at the pulse energy of 180 µJ, one may notice the selectivity of the radiation sintering process: some NPs remained unmodified and represent initial agglomerates (Figure 6). For example, such selectivity is clearly observed in TEM images of NPs sintered by laser radiation with the wavelength of 527 nm at the high gas flow rates. In this respect, the thermal sintering method is not selective and is more suitable for the problems of obtaining spherical NPs, regardless of their optical absorbing properties.

## 5. Conclusions

This work demonstrates the method for modifying aerosol gold NPs by nanosecond pulsed-periodic laser radiation with wavelengths of 527 and 1053 nm, combining the radiation direction with the aerosol flow. Primary NPs with the characteristic sizes of about 10 nm synthesized by the SD directly in gas stream formed dendrite-like agglomerates with the average sizes of 188–280 nm during transportation, depending on the gas flow rate. Since agglomerates formed in the flow were spatially separated and their interaction with each other and with the walls of the experimental cell was insignificant, it was possible to determine the extinction cross-section of a single NP averaged over the flow. From these spectra, it was found that the agglomerates extinction varied slightly in the wavelength range of 350–1000 nm. For thermally modified agglomerates in the flow with a spherical shape, the extinction cross-section increased with an increase in the gas flow rate and demonstrated a plasmon absorption peak at the wavelength of about 528 nm.

In two series of experiments on the modification of aerosol NPs by nanosecond pulsed laser radiation, we studied on-line the dynamics of changes in the NPs size at the different gas flows while varying the pulse energy and number of laser radiation pulses. The decrease in the NPs size with the increase in the energy of laser pulses was characterized by an S-shaped shrinkage curve with a rapid size reduction in the region of a certain threshold pulse energy. Such a curve was found to be typical for the sintering of powder green bodies. Initial agglomerates transformed into spherical NPs at the laser pulse energies above the threshold, which was further confirmed by NPs TEM images. It is important to emphasize that the pulse energy and efficiency of radiation absorption by NPs turned out to be the key parameters for NPs laser modification compared to laser pulse repetition rate and average radiation power. At the laser pulse energies above the threshold, the complete sintering of aerosol agglomerates with subsequent transformation into spherical NPs could be realized by the minimum required number of pulses, which depends only on their energy. For instance, the complete sintering of agglomerates by pulsed radiation with the wavelength of 1053 nm at the pulse energy of 900 µJ was achieved by six pulses. At the same time, the further effect of laser pulses on NPs turned out to be excessive, since the acquired spherical shape no longer changed.

Another peculiarity of NPs sintering by nanosecond laser pulses was the pulse-periodic thermal mode. It was characterized by a short nanosecond time of the heated state after the pulse impact and a long millisecond period of time at the ambient gas temperature before the arrival of the next pulse. In this case, the discrete-stepwise process of NPs sintering was realized, which was stimulated by a number of consecutive pulses. Due to this, the behavior of laser-modified NPs corresponded to the temperature of the surrounding gas, i.e., it was characterized by low Brownian motion velocities, which significantly reduced the effect of NPs secondary agglomeration.

In the future, it would be of interest to study the sintering processes of aerosol NPs under the influence of subnanosecond laser radiation pulses, which implement an almost adiabatic mode of energy input, since the cooling time of gold NPs in argon is in order of 100 ns. The expected effect may be NPs’ complete sintering at a significantly reduced threshold value of pulse energy.

## Figures and Tables

**Table 1:** Extinction cross-sections of laser-modified NPs, initial agglomerates, and thermally modified NPs at the wavelength of 1053 nm.

Aerosol Flow Rate, mL/min	$\sigma_{LM},\;µm^{2}$	$\sigma_{A},\;µm^{2}$	$\sigma_{S},\;µm^{2}$
50	0.005	0.08	0.02
100	0.006	0.08	0.02
200	0.006	0.09	0.04
400	0.004	0.10	0.09

**Table 2:** Extinction cross-sections of laser-modified NPs, initial agglomerates, and thermally modified NPs at the wavelength of 527 nm.

Aerosol Flow Rate, mL/min	$\sigma_{LM},\;µm^{2}$	$\sigma_{A},\;µm^{2}$	$\sigma_{S},\;µm^{2}$
50	0.04	0.06	0.07
100	0.05	0.06	0.07
200	0.12	0.07	0.13
400	0.16	0.09	0.27
